# Trends in the effects of socioeconomic position on physical activity levels and sedentary behavior among Korean adolescents

**DOI:** 10.4178/epih.e2023085

**Published:** 2023-09-08

**Authors:** Hunju Lee, Hyowon Choi, Sang Baek Koh, Hyeon Chang Kim

**Affiliations:** 1 Department of Preventive Medicine, Yonsei University Wonju College of Medicine, Wonju, Korea; 2 Department of Public Health, Yonsei University Graduate School, Seoul, Korea; 3 Department of Preventive Medicine, Yonsei University College of Medicine, Seoul, Korea; 4 Institute for Innovation in Digital Healthcare, Yonsei University Health System, Seoul, Korea

**Keywords:** Exercise, Sedentary behavior, Social class, Adolescent, Korea Youth Risk Behavior Survey

## Abstract

**OBJECTIVES:**

We examined trends in physical activity and sedentary behavior in Korean adolescents, and their association with socioeconomic position (SEP).

**METHODS:**

We used data from the Korea Youth Risk Behavior Survey, a nationwide study involving students aged 12-19 conducted between 2009 and 2021. SEP was assessed based on economic status, parental education attainment, and urbanization. Physical activity was categorized into vigorous physical activity, moderate physical activity, and muscle training, and sedentary time was also measured. We conducted the log-binomial regression to calculate prevalence ratios (PRs) and prevalence differences.

**RESULTS:**

Our analysis included a total of 593,896 students. We observed an increasing trend in physical activity, but a worsening trend in sedentary behavior. A positive association was found between an adolescent’s physical activity and SEP indicators, except for urbanization. Adolescents with higher economic status engaged in more vigorous physical activity (high: PR, 1.26; 95% confidence interval [CI], 1.25 to 1.28; middle: PR, 1.03; 95% CI, 1.02 to 1.04). Similar associations were observed for father’s education (tertiary or above: PR, 1.11; 95% CI, 1.09 to 1.13; upper secondary: PR, 1.05; 95% CI, 1.03 to 1.07) and mother’s education (tertiary or above: PR, 1.17; 95% CI, 1.15 to 1.20; upper secondary: PR, 1.06; 95% CI, 1.04 to 1.08). Adolescents with higher economic status also showed a higher compliance rate with the guideline restricting sedentary time to 2 hours per day (high: PR, 1.28; 95% CI, 1.25 to 1.30; middle: PR, 1.03; 95% CI, 1.01 to 1.05).

**CONCLUSIONS:**

Adolescents with higher SEP exhibited more physical activity and less sedentary time than those with lower SEP.

## GRAPHICAL ABSTRACT

[Fig f4-epih-45-e2023085]

## INTRODUCTION

Physical activity is a fundamental health behavior that is particularly crucial for children and adolescents. In this age group, physical activity is associated with various health outcomes, including adiposity, blood pressure, cardiorespiratory fitness, mental health, cognitive function, and academic performance [1]. However, despite the importance of physical activity, recent studies have highlighted the insufficient levels of physical activity among the majority of adolescents worldwide. For instance, the World Health Organization (WHO) reported that 81% of adolescents spent less than 1 hour a day in moderate-to-vigorous physical activity [2]. In Korea, specifically, only 5.9% of adolescents meet the physical activity guideline [3], and just 19.3% meet the aerobic exercise guideline [4].

Several factors influence an individual’s level of physical activity, including demographics (e.g., age, sex, and health status), environment factors (e.g., availability of facilities), and national policies. Among these factors, socioeconomic position (SEP) plays a critical role. Numerous previous papers have demonstrated a positive association between higher SEP and physical activity [5,6]. However, the relationship between SEP and physical activity in adolescents may vary based on sex, race/ethnicity, and geographic location. Notably, studies conducted in Nordic countries reported weaker associations between SEP and physical activity compared to studies in the United States, likely due to cultural differences [7].

Despite the low prevalence of physical activity among Korean adolescents, there is a lack of research examining the impact of SEP on their physical activity levels. While a study using data from the Korean National Health and Nutrition Examination Survey (KNHANES) reported a positive association between high SEP and physical activity prevalence in Korean adolescents in 2013, it had limitations, including the use of a limited SEP index (e.g., economic status and area of residence) and a small, less representative sample of Korean adolescents, as the KNHANES is not specifically designed for the age group [8].

Therefore, the aim of this study was to investigate the trends in physical activity among Korean adolescents, explore the association between SEP and physical activity, and analyze trends in inequality in physical activity using the Korea Youth Risk Behavior Survey (KYRBS), a nationwide survey representative of Korean adolescents.

## MATERIALS AND METHODS

### Study population

We utilized data from the KYRBS, a nationally representative web-based survey conducted annually since 2005 by the Korea Disease Control and Prevention Agency (KDCA) and the Ministry of Education [9]. The survey captures various health behaviors among Korean adolescents, including smoking, drinking, obesity, and physical activity. The KYRBS employs a 3-step sampling process, involving population stratification by regional and school characteristics, sampling distribution by proportional allocation (selecting 400 middle schools and 400 high schools), and sampling using the stratified cluster method (school and class units). The participant response rates for the survey ranged from 92.9% to 97.7% from 2009 to 2021.

For our analysis, we included data from 2009 to 2021, as the questionnaire items related to physical activity changed in 2009. The total population consisted of 865,614, and we excluded 271,718 participants with missing data (age: 4,911; sedentary time: 23,219; father’s education: 209,293; mother’s education: 202,076; there were no missing data for other variables). Finally, our analysis included 593,896 participants. We also conducted a sensitivity analysis for excluded participants.

### Variables

We selected 4 variables as indicators of SEP: perceived household economic status, father’s education attainment, mother’s education attainment, and urbanization. Economic status was collected by participants’ subjective selection of 1 of 5 groups (high, upper-middle, middle, lower-middle, and low) when asked about the household economic status. Father’s and mother’s education attainment was classified into 3 groups (middle school or lower, high school, and college or higher), and this information was only collected for participants who lived with their parents. We separately analyzed the effect of father’s and mother’s education attainment, but also presented the effect of the parent’s education attainment using the higher of the two. Urbanization was divided into 3 groups based on administrative districts (metropolitan, other cities, and rural areas).

We examined three physical activity outcomes: vigorous physical activity (VPA), moderate physical activity (MPA), and muscle training. VPA was measured by the number of days on which the participant engaged in activities that caused heavy sweating or a large increase in breathing or heart rate, such as jogging, soccer, or baseball, for more than 20 minutes in the past 7 days. Participants who engaged in VPA for at least 3 days a week were classified as having sufficient VPA. MPA was measured by the number of days on which the participant engaged in activities that caused only light sweating or a slight to moderate increase in breathing or heart rate, regardless of the type of activity, for more than 60 minutes a day. Participants who engaged in MPA for at least 5 days a week were classified as having sufficient MPA. Muscle training was measured by the number of days on which the participant engaged in muscle training exercises, such as push-ups, dumbbell exercises, or pull-up bar exercises. Participants who engaged in muscle training for at least 3 days a week were classified as having sufficient muscle training. We followed the KYRBS user’s guide on physical activity for this categorization.

The measurement of sedentary time changed in 2013. Prior to 2013, sedentary time was measured by the mean hours of sedentary behavior per day for recreational purposes on weekdays and weekends, separately. Starting in 2013, sedentary time was measured based on the mean hours of sedentary behavior per day for each purpose and time. To evaluate sedentary time, we used the American Academy of Pediatrics’ guideline [10]. Participants who had less than 2 hours a day of sedentary time on weekdays for recreational purposes were classified as having an appropriate level of sedentary time. We also included sex and school type (middle and high school) for subgroup analysis.

### Statistical analysis

We conducted a descriptive analysis to present participants’ basic characteristics. Age was presented as mean and standard deviation, and categorical variables were presented as proportion (%). We used the weights provided by the KDCA to account for the complex sampling design.

To examine the trends in physical activity and sedentary time, we employed design-corrected binomial regression analysis [11]. To account for the effects of the coronavirus disease 2019 (COVID-19) pandemic and survey question changes, we used data from 2009 to 2018 to test trends in VPA, and data from 2013 to 2019 to test trends in sedentary time. To test trends in MPA and muscle training, data from 2009 to 2019 were used. Trend testing was also performed for the entire period (2009 to 2021) of each physical activity variable (Supplementary Material 1).

We used the prevalence ratio (PR) and prevalence difference (PD) to analyze differences in physical activity according to SEP. The PR was calculated using a log-binomial model. All PR and PD values were weighted. We presented both the total and yearly PR values. Given that the percentage of participants with missing data for parents’ education was 20%, we conducted a sensitivity analysis to reduce the bias from the missing data. First, we reanalyzed the other SEP variables (economic status and urbanization) including the participants with missing responses on parents’ education, as there were no missing data for economic status and urbanization. Second, we included the participants for whom only 1 parent’s education was known in a sensitivity analysis for parents’ education, unlike the main analysis, which was limited to participants who reported both parents’ educational status. Parents’ education was analyzed separately according to the higher educational attainment of the two (Supplementary Material 2). Additionally, we conducted subgroup analyses by sex and school type (Supplementary Material 3). The yearly PR values and confidence intervals (CIs) are presented in Supplementary Material 4.

All statistical analyses were performed using R version 4.2.2 (R Foundation for Statistical Computing, Vienna, Austria) with the ‘survey’ package [12]. Statistical significance was considered at a p-value < 0.05.

### Ethics statement

The study protocol was approved by the Institutional Review Board (IRB) of Wonju Severance Christian Hospital (IRB No. CR323315). The requirement for informed consent was waived by the IRB.

## RESULTS

Table 1 presents the basic characteristics of participants and trends in physical activity from 2009 to 2021 among Korean adolescents. A total of 593,896 students were included in the analysis. All kinds of SEP showed a decrease in the less-privileged group, except for urbanization. The percentage of those who engaged in VPA slightly increased from 31.0% in 2009 to 37.9% in 2018, but dropped to 26.9% in 2020 before recovering to 29.4% in 2021. The proportions of participants who engaged in MPA and muscle training showed slight increases from 10.7% to 14.2% and from 19.7% to 21.2%, respectively. The compliance rate for sedentary time decreased from 56.4% in 2009 to 20.9% in 2021.

**Figure 1.**
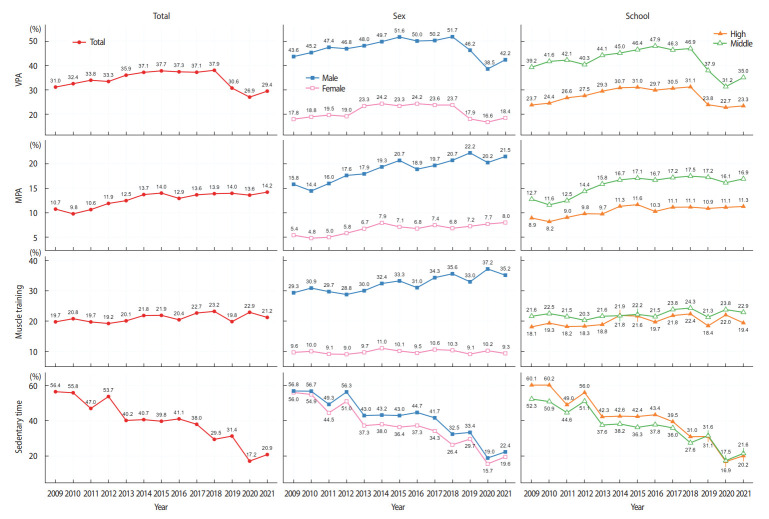
Trends in the prevalence of physical activity and sedentary time in Korean adolescents during 2009-2021. The lines in the figure represent the rates of compliance with guidelines for physical activity and sedentary time. The guidelines recommend vigorous physical activity (VPA) for at least 20 minutes a day, 3 days a week; moderate physical activity (MPA) for at least 60 minutes a day, 5 days a week; muscle training 3 days a week; and a maximum of 2 hours of sedentary time per day on weekdays for recreational purposes.

Figure 1 displays trends in physical activity and sedentary time among Korean adolescents by sex and school type. The prevalence of physical activity among male participants was 3 times higher than that among female participants for all categories. The overall trends in each subgroup were similar to those in the total sample. From 2009 to 2018, an upward trend was observed in VPA, as well as from 2009 to 2019 for MPA and muscle training (p<0.01). Although the rate of muscle training increased in boys, there was no significant change in the trend for girls. The prevalence of sedentary time decreased significantly in all subgroups 2013 to 2019 (p<0.01; Supplementary Material 1).

**Figure 2.**
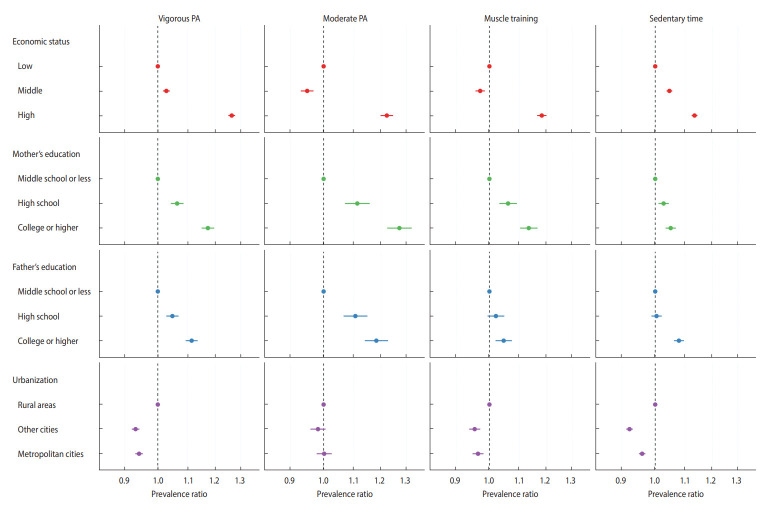
The overall prevalence ratios of Korean adolescents’ socioeconomic position and physical activity (PA). Dots and lines mean estimates and 95% confidence intervals of prevalence ratios. The reference groups are low for economic status, middle school or less for mother’s education and father’s education, and rural areas for urbanization. The x-axis is shown on a log-scale.

Table 2 and Figure 2 present the weighted PRs of physical activity and sedentary time. Economic status, father’s education, and mother’s education were significantly associated with physical activity and sedentary time. However, middle-class status had a negative association with MPA (PR, 0.95; 95% CI, 0.93 to 0.97) and muscle training (PR, 0.96; 95% CI, 0.95 to 0.98). Moreover, urbanization had adverse associations with physical activity and sedentary time. The sensitivity analysis, which included the excluded participants, and the subgroup analysis showed similar results (Supplementary Materials 2 and 3).

**Figure 3.**
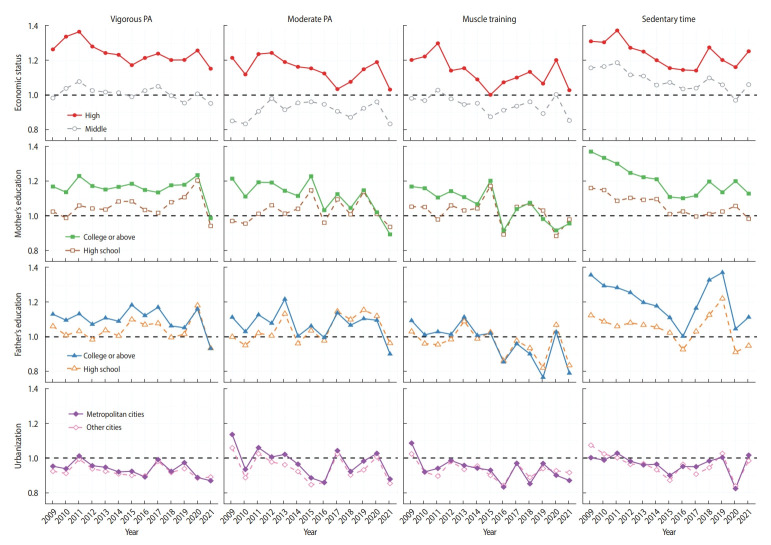
Trends in the prevalence ratios for Korean adolescents’ socioeconomic position and physical activity (PA). Dots represent estimates of the prevalence ratio for each year. The reference groups are low for economic status, middle school or less for mother’s education and father’s education, and rural areas for urbanization. All the estimates and 95% confidence intervals can be found in Supplementary Material 4.

This association was consistently observed across all years (Figure 3), with the effect being more pronounced in the higher SEP groups (such as those with a “high” economic status and “tertiary or above” education). There was little change in the PRs until 2019, despite some fluctuations. However, the COVID-19 pandemic triggered a dramatic increase in the PRs in 2020, which subsequently recovered in 2021. Supplementary Material 4 contains the point estimates and CIs.

Table 3 presents the PDs of physical activity and sedentary time among Korean adolescents according to their SEP. The results indicated that economic status, father’s education, and mother’s education were positively associated with physical activity and negatively associated with sedentary time. Meanwhile, urbanization had an inverse association with physical activity. However, middle-class economic status showed no significant association with physical activity.

## DISCUSSION

In this study, we observed several key findings. First, despite a minor upward trend in physical activity among adolescents, the overall rate of physical activity remains very low, particularly among girls. Additionally, we noted a concerning decrease in the compliance rate with the guideline on sedentary behavior. Second, all SEP indicators, except urbanization, showed positive associations with adolescent physical activity and sedentary time. Third, there was a slight decreasing trend in PRs over time. However, the prevalence of physical activity and sedentary time have been significantly impacted by COVID-19, emphasizing the need for continuous monitoring.

It is notable that only a small proportion of Korean adolescents, especially girls, engaged in the recommended amount of physical activity. As shown in Table 1 and Figure 1, despite a modest increase, the prevalence of sufficient MPA and VPA remained 14.2% and 29.4%, respectively. As a comparison with global figures, 81% of adolescents engaged in insufficient physical activity in 2016, versus 76.3% in Singapore, 84.3% in China, and 79.4% in high-income countries [13]. Our results indicate that the prevalence of sufficient physical activity among Korean adolescents is lower than in similar high-income countries and neighboring East Asian countries.

Several factors contribute to the low prevalence of adequate physical activity among Korean adolescents. One significant factor is time constraints. Korea has some of the longest study hours worldwide, with middle school students averaging 5 hours and 57 minutes per day, and high school students averaging 6 hours and 44 minutes per day in 2019 [14]. This is in stark contrast to American adolescents, who spent an average of only 3 hours and 30 minutes per day studying in the same year [15]. Furthermore, social relationships and the built environment can also impact adolescent physical activity levels. The 2022 Report Card Korea for Global Matrix 4.0 indicates that adherence to the WHO guideline among parents of children and adolescents aged 12 years to 18 years was 36.9% and 46.1%, respectively [16]. The report also highlighted issues such as air pollution and a lack of green space in Korea.

All types of physical activities demonstrated slight increases until 2019. The prevalence of MPA increased by 3.3%p for the total population (from 10.7% in 2009 to 14.0% in 2019), 5.7%p for boys (from 15.8 to 21.5%), and 2.6%p for girls (from 5.4%p to 8.0%). The patterns of physical activity among Korean adolescents exhibited similarities to global trends observed from 2001 to 2016, with boys showing a decrease of 2.5%p in physical inactivity (from 80.1 to 77.6%) and girls experiencing a slight decrease of 0.4%p (from 85.1 to 84.7%) [13].

Conversely, the compliance rate with the sedentary time guideline showed an opposite trend, decreasing by 25%p over a 10-year period, from 56.4% in 2009 to 31.4% in 2019. This finding is similar to the dramatic increase in sedentary behavior observed in other countries [17,18]. The proliferation of digital devices such as smartphones and personal computers over the past two decades has contributed to the rise in sedentary behavior among adolescents globally. For example, Twenge et al. [19] reported that daily digital media usage among United States adolescents escalated from 5.03 hours in 2006 to 7.42 hours in 2016. Similar trends were observed in a multi-country study on computer use [20]. In Korea, Internet usage among adolescents increased by about an hour, from 3 hours and 20 minutes in 2016 to 4 hours and 30 minutes in 2019, representing a 1.8-fold increase over the past 2 years. Sedentary behavior can lead to various health problems due to insufficient physical activity [21], and can cause problems such as depression and psychological distress [22]. Therefore, the increasing prevalence of sedentary behavior among adolescents raises concerns.

Upon analyzing data spanning 13 years, from 2009 to 2021, it appears that higher SEP correlates with increased physical activity among adolescents (Table 2). Although the implications of each indicator may shift over time, necessitating careful interpretation, this finding remains significant in summarizing the impact of SEP on youth physical activity within our society. Consistent findings were also observed in the annual analysis (Supplementary Material 4). This trend also appeared in the PD values (Table 3), which showed similar figures to Denmark [23] and the United Kingdom [24].

To understand the various effects of SEP, we utilized 4 indicators (economic status, father’s education, mother’s education, and urbanization). Except for urbanization, all indicators showed positive associations with physical activity among adolescents, which is consistent with previous studies. For instance, a systematic review of 62 articles on adolescent SEP and physical activity found that adolescents with higher SEP were more physically active than those with lower SEP [7]. Other studies using the KNHANES [8] and indicators such as family affluence score [25], have reported similar findings among Korean adolescents.

Previous studies have often employed ecological models to explain how SEP affects adolescent physical activity by categorizing the factors into intrapersonal, social, and environmental. For example, Humbert et al. [26] examined the differences between high-SEP and low-SEP adolescents using an ecological model. The study found that high-SEP adolescents cited time barriers due to extracurricular activities as an intrapersonal factor, parental involvement in driving to facilities as a social factor, and a diverse of physical programs as environmental factors. In contrast, low-SEP adolescents reported time barriers due to family obligations as an intrapersonal factor, adult involvement (e.g., coaches) as a social factor, and proximity, cost of facilities and safety as environmental factors.

Additionally, Yoo et al. [27] conducted focus group interviews with Korean adolescents from low-income families to identify barriers to physical activity. The study found that Korean adolescents with low SEP identified time barriers due to studying as the most important factor. They also highlighted the importance of knowledge about the benefits of physical activity, encouragement from friends and adults, and diverse sports programs based on schools. These results indicate that various social and environmental factors, in addition to direct financial effects, influence adolescent physical activity.

While other SEP indicators showed clear associations with adolescent physical activity, urbanization demonstrated an inverse effect. Previous studies have reported inconsistent results regarding urbanization [28-31]. There are several possible explanations for this phenomenon. First, it is the built environment, rather than urbanization itself, that primarily affects people’s physical activity. Sallis et al. [32] argued that multilevel interventions, social environments, physical environments, and police are required to achieve population-level changes in physical activity. International studies have shown that the built environment, including parks, public transportation, and residential density, is associated with physical activity [33,34]. This association extends to adolescents as well [35]. Another explanation is that urbanization has different effects on the different types of physical activity. For instance, a study showed that children in rural areas engaged in more outdoor activities but less structured physical activity compared to urban children [36]. Furthermore, the timing of exercise also plays a role in these results. Huang et al. [37] demonstrated that urban children engaged in more physical activity after school, on holidays, and on weekends than rural children. Therefore, it is necessary to consider complex factors when interpreting the effect of urbanization on adolescent physical activity.

The COVID-19 pandemic significantly impacted the physical activity levels of Korean adolescents. In 2020, when the pandemic broke out, the Korean government implemented social distancing policies such as lockdowns, closure of public facilities, and closure of schools. Schools remained closed for 2 months in March 2020 and April 2020, and even after reopening, online classes were conducted for a year. Physical education classes mainly focused on home training instead of team sports, considering the limitations of online classes and the restrictions during the pandemic [38]. This change influenced the physical activity levels of adolescents, resulting in a decrease in the prevalence of VPA and MPA, but an increase in the prevalence of muscle training. Additionally, the decrease in public sports classes amplified the relative effect of extracurricular sports. This phenomenon, where physical activity was more affected in low-SEP groups, has also been observed in other countries [39,40].

The prevalence of VPA recovered to 29.4% in 2021 with the reopening of schools, but the prevalence of MPA remained low (Table 1). This discrepancy can be attributed to the fact that VPA, which requires more than 20 minutes per day and 3 days a week, can be easily achieved through school physical education classes, whereas MPA, which requires more than 60 minutes every day, requires more social resources, such as public gyms and parks, that were still closed in 2021. The compliance rate with the sedentary time guideline also dropped to 17.2% in 2020, but recovered to 20.9% in 2021. It is expected that the prevalence of physical activity will increase in 2022 as social distancing ends. The PRs in 2021 also showed a dramatic decrease in almost all outcomes. This was because the physical activity of the low-SEP group had recovered to pre-COVID-19 pandemic levels, while the physical activity of the moderate-SEP and high-SEP groups had not.

Our study has several strengths. First, we utilized weighted data from the KYRBS, a nationwide survey representing Korean adolescents. Second, we analyzed 13 years of data from 2009 to 2021, enabling us to obtain more precise results and analyze trends in physical activity. Moreover, our study period included the COVID-19 pandemic period, allowing us to examine the effects of COVID-19. Lastly, we used various indicators to measure SEP, which facilitated an exploration of differences in their effects.

However, our study also has some limitations. First, the variables for physical activity, sedentary time, and SEP were self-reported, which may have introduced misclassification bias, particularly considering that the respondents were adolescents. Secondly, there were multiple changes in the questionnaire over time. The questionnaire for VPA was changed in 2019, and that for sedentary time was modified in 2013. These changes made it challenging to interpret trends accurately. Additionally, the investigation method changed in 2021, from using only personal computers to using both personal computers and tablet computers. Third, consent for sensitive questions such as ethnicity and parents’ education were added in 2019, which reduced the response rate for parents’ education. However, sensitivity analyses were conducted, and the results showed similar outcomes.

In conclusion, our study reveals a low prevalence of sufficient physical activity and high sedentary time in Korean adolescents. We found that higher economic status, father’s education, and mother’s education were associated with more physical activity and less sedentary time among adolescents. Therefore, interventions that promote physical activity and reduce the SEP gap are necessary.

## Figures and Tables

**Figure f4-epih-45-e2023085:**
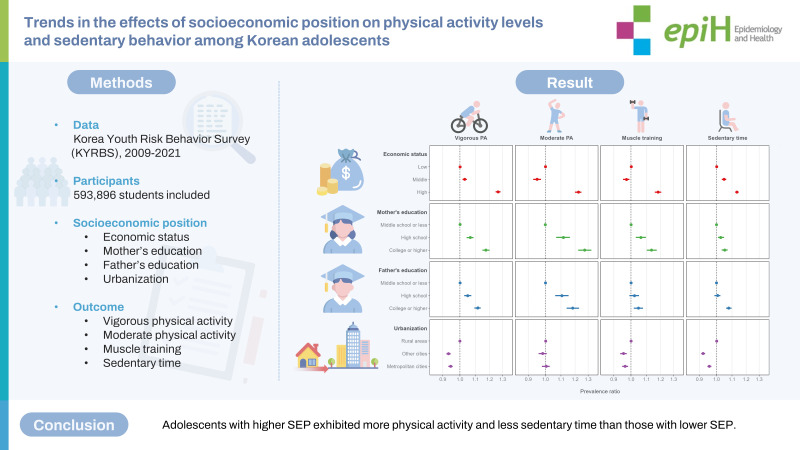


**Table 1.** Characteristics of the study population from 2009 to 2021

Characteristics		Year												
		2009	2010	2011	2012	2013	2014	2015	2016	2017	2018	2019	2020	2021
Total (n)		57,447	55,731	58,468	56,732	51,534	51,009	47,742	46,062	43,294	40,672	26,152	29,658	29,395
Age, mean (yr)		15.21	15.24	15.26	15.06	15.11	15.15	15.21	15.25	15.26	15.28	15.05	15.19	15.19
Sex														
	Boys	51.2	51.6	51.4	51.5	51.1	50.5	50.8	50.8	50.8	50.9	44.9	47.0	46.0
	Girls	48.8	48.4	48.6	48.5	48.9	49.5	49.2	49.2	49.2	49.1	55.1	53.0	54.0
Stage														
	High school	52.6	53.3	53.4	54.2	55.4	55.5	56.4	58.4	58.1	56.7	51.4	50.5	48.1
	Middle school	47.4	46.7	46.6	45.8	44.6	44.5	43.6	41.6	41.9	43.3	48.6	49.5	51.9
Economic status														
	Low	21.4	20.6	19.5	19.6	17.6	15.4	14.2	12.9	11.5	10.6	10.2	10.2	8.7
	Middle	47.8	46.6	47.1	46.9	46.9	47.5	46.1	46.4	44.2	44.3	46.0	45.4	46.1
	High	30.8	32.7	33.4	33.5	35.5	37.1	39.7	40.7	44.3	45.1	43.9	44.4	45.2
Father’s education														
	Middle school or less	6.3	5.8	4.9	4.3	3.8	3.1	2.7	2.3	2.0	1.6	1.6	1.5	1.3
	High school	42.5	40.7	39.9	40.1	39.1	36.1	34.0	33.7	31.3	29.3	28.0	26.4	24.9
	College or higher	51.2	53.5	55.3	55.5	57.0	60.8	63.3	64.0	66.6	69.1	70.4	72.1	73.8
Mother’s education														
	Middle school or less	6.5	5.9	4.9	4.4	3.7	2.7	2.3	2.0	1.7	1.4	1.3	1.1	1.0
	High school	56.5	54.8	53.2	52.2	50.2	46.5	43.0	41.4	38.2	34.8	32.5	30.6	28.5
	College or higher	37.0	39.3	41.9	43.4	46.1	50.8	54.6	56.6	60.1	63.9	66.1	68.4	70.4
Urbanization														
	Rural areas	5.0	6.0	5.7	5.8	6.5	6.0	5.7	5.1	5.6	5.5	5.2	5.5	4.8
	Other cities	40.1	48.2	48.8	49.2	48.6	49.8	50.0	50.9	50.3	50.8	51.0	51.3	53.0
	Metropolitan cities	54.9	45.8	45.5	45.0	44.8	44.2	44.4	44.1	44.1	43.7	43.8	43.2	42.3
Guideline compliance rate for PA and sedentary time														
	Vigorous PA	31.0	32.4	33.8	33.3	35.9	37.1	37.7	37.3	37.1	37.9	30.6	26.9	29.4
	Moderate PA	10.7	9.8	10.6	11.9	12.5	13.7	14.0	12.9	13.6	13.9	14.0	13.6	14.2
	Muscle training	19.7	20.8	19.7	19.2	20.1	21.8	21.9	20.4	22.7	23.2	19.8	22.9	21.2
	Sedentary time	56.4	55.8	47.0	53.7	40.2	40.7	39.8	41.1	38.0	29.5	31.4	17.2	20.9

Values are presented as %. PA, physical activity.

**Table 2.** The prevalence ratio between socioeconomic position (SEP) and physical activity (PA) (n=593,896)^1^

SEP		Vigorous PA	Moderate PA	Muscle training	Sedentary time
Economic status					
	Low	1.00 (reference)	1.00 (reference)	1.00 (reference)	1.00 (reference)
	Middle	1.03 (1.02, 1.04)	0.95 (0.93, 0.97)	0.97 (0.96, 0.99)	1.03 (1.01, 1.05)
	High	1.26 (1.25, 1.28)	1.22 (1.20, 1.25)	1.18 (1.16, 1.20)	1.28 (1.25, 1.30)
Father’s education					
	Middle school or less	1.00 (reference)	1.00 (reference)	1.00 (reference)	1.00 (reference)
	High school	1.05 (1.03, 1.07)	1.11 (1.07, 1.15)	1.02 (0.99, 1.05)	0.96 (0.93, 0.99)
	College or higher	1.11 (1.09, 1.13)	1.18 (1.14, 1.23)	1.05 (1.02, 1.07)	1.15 (1.12, 1.19)
Mother’s education					
	Middle school or less	1.00 (reference)	1.00 (reference)	1.00 (reference)	1.00 (reference)
	High school	1.06 (1.04, 1.08)	1.11 (1.07, 1.16)	1.06 (1.03, 1.09)	1.01 (0.98, 1.04)
	College or higher	1.17 (1.15, 1.20)	1.27 (1.22, 1.32)	1.13 (1.10, 1.16)	1.15 (1.11, 1.19)
Urbanization					
	Rural areas	1.00 (reference)	1.00 (reference)	1.00 (reference)	1.00 (reference)
	Other cities	0.93 (0.92, 0.94)	0.98 (0.96, 1.01)	0.95 (0.94, 0.97)	0.91 (0.89, 0.93)
	Metropolitan cities	0.94 (0.93, 0.95)	1.00 (0.98, 1.03)	0.96 (0.95, 0.98)	1.00 (0.98, 1.02)

Values are presented as prevalence ratio (95% confidence interval); All statistics were weighted.

1 Each value for PA and sedentary time corresponds to the guideline compliance rate; The guidelines recommend vigorous PA for at least 20 minutes a day, 3 days a week; moderate PA for at least 60 minutes a day, 5 days a week; muscle training 3 days a week; and a maximum of 2 hours of sedentary time per day on weekdays for recreational purposes.

**Table 3.** Prevalence differences between socioeconomic position (SEP) and physical activity (PA) (n=593,896)^1^

SEP		Vigorous PA	Moderate PA	Muscle training	Sedentary time
Economic status					
	Low	Reference	Reference	Reference	Reference
	Middle	0.9 (0.5, 1.2)	-0.6 (-0.8, -0.4)	-0.6 (-0.9, -0.3)	0.5 (0.2, 0.7)
	High	8.2 (7.9, 8.6)	2.7 (2.4, 2.9)	3.6 (3.3, 3.9)	4.1 (3.8, 4.4)
Father’s education					
	Middle school or less	Reference	Reference	Reference	Reference
	High school	1.5 (0.9, 2.1)	1.2 (0.7, 1.6)	0.4 (-0.1, 1.0)	-0.6 (-1.1, -0.1)
	College or higher	3.6 (3.0, 4.2)	2.0 (1.6, 2.4)	0.9 (0.4, 1.5)	2.3 (1.9, 2.8)
Mother’s education					
	Middle school or less	Reference	Reference	Reference	Reference
	High school	2.0 (1.3, 2.6)	1.2 (0.8, 1.6)	1.2 (0.6, 1.7)	0.1 (-0.3, 0.6)
	College or higher	5.3 (4.7, 6.0)	2.9 (2.5, 3.3)	2.6 (2.0, 3.1)	2.3 (1.8, 2.8)
Urbanization					
	Rural areas	Reference	Reference	Reference	Reference
	Other cities	-2.5 (-2.9, -2.1)	-0.2 (-0.5, 0.1)	-1.0 (-1.4, -0.6)	-1.5 (-1.8, -1.2)
	Metropolitan cities	-2.1 (-2.6, -1.7)	0.0 (-0.3, 0.3)	-0.8 (-1.2, -0.4)	0.1 (-0.3, 0.4)

Values are presented as prevalence difference % (95% confidence interval); All statistics were weighted.

1 Each value for PA and sedentary time corresponds to the guideline compliance rate; The guidelines recommend vigorous PA for at least 20 minutes a day, 3 days a week; moderate PA for at least 60 minutes a day, 5 days a week; muscle training 3 days a week; and a maximum of 2 hours of sedentary time per day on weekdays for recreational purposes.

## SUPPLEMENTARY MATERIALS

Supplement Material 1. The estimates and p-values for trend test

Supplement Material 2. The prevalence ratio between socio-economic position and physical activity

Supplement Material 3. Subgroup analysis for sex and school

Supplement Material 4. The yearly prevalence ratio between socioeconomical position and physical activity in Korean adolescents
