# Supplementary material for: Trends in the effects of socioeconomic position on physical activity levels and sedentary behavior among Korean adolescents
Source: Epidemiol Health. 2023 Sep 8;45:e2023085. doi: 10.4178/epih.e2023085 (PMC10728613; doi:10.4178/epih.e2023085)
Supplement: Supplement Material 1. — The estimates and p-values for trend test [file epih-45-e2023085-Supplementary-1.docx]

Supplementary Material 1. The estimates and p-values for trend test

|  | Total | | Sex | | | |  | School | | | |
| --- | --- | --- | --- | --- | --- | --- | --- | --- | --- | --- | --- |
| Variables |  |  | Male | | Female | |  | High | | Middle | |
|  | Est. | P-value | Est. | P-value | Est. | P-value |  | Est. | P-value | Est. | P-value |
| Vigorous PA |  |  |  |  |  |  |  |  |  |  |  |
| 2009 ~ 2018 | 0.67 | < 0.01 | 0.66 | < 0.01 | 0.86 | < 0.01 |  | 0.83 | < 0.01 | 0.71 | < 0.01 |
| 2009 ~ 2021 | 0.02 | 0.76 | 0.16 | 0.01 | 0.19 | 0.01 |  | 0.18 | 0.06 | -0.15 | 0.04 |
| Moderate PA |  |  |  |  |  |  |  |  |  |  |  |
| 2009 ~ 2019 | 0.80 | < 0.01 | 0.91 | < 0.01 | 0.92 | < 0.01 |  | 0.67 | < 0.01 | 0.95 | < 0.01 |
| 2009 ~ 2021 | 0.86 | < 0.01 | 1.02 | < 0.01 | 1.07 | < 0.01 |  | 0.72 | < 0.01 | 0.95 | < 0.01 |
| Muscle training |  |  |  |  |  |  |  |  |  |  |  |
| 2009 ~ 2019 | 0.29 | < 0.01 | 0.54 | < 0.01 | 0.13 | 0.13 |  | 0.44 | < 0.01 | 0.15 | 0.08 |
| 2009 ~ 2021 | 0.29 | < 0.01 | 0.67 | < 0.01 | 0.05 | 0.54 |  | 0.38 | < 0.01 | 0.19 | 0.03 |
| Sedentary time |  |  |  |  |  |  |  |  |  |  |  |
| 2013 ~ 2019 | -0.94 | < 0.01 | -0.89 | < 0.01 | -0.93 | < 0.01 |  | -1.16 | < 0.01 | -0.66 | < 0.01 |
| 2009 ~ 2021 | -2.87 | < 0.01 | -2.65 | < 0.01 | -3.07 | < 0.01 |  | -3.15 | < 0.01 | -2.57 | < 0.01 |
