# Supplementary material for: Trends in the effects of socioeconomic position on physical activity levels and sedentary behavior among Korean adolescents
Source: Epidemiol Health. 2023 Sep 8;45:e2023085. doi: 10.4178/epih.e2023085 (PMC10728613; doi:10.4178/epih.e2023085)
Supplement: Supplement Material 2. — The prevalence ratio between socio-economic position and physical activity [file epih-45-e2023085-Supplementary-2.docx]

Supplementary Material 2. The prevalence ratio between socio-economic position and physical activity

| Variables | | | Vigorous PA | | | | Moderate PA | | | Muscle training | | | | Sedentary time | | |  |
| --- | --- | --- | --- | --- | --- | --- | --- | --- | --- | --- | --- | --- | --- | --- | --- | --- | --- |
|  |  |  | PR (95% CI) | | | | PR (95% CI) | | | PR (95% CI) | | | | PR (95% CI) | | |  |
| Household income (n=865,614) | |  | |  |  |  | |  |  | |  |  |  | |  |  | |
|  | Low | | (reference) | | | | (reference) | | | (reference) | | | | (reference) | | |  |
|  | Middle | | 1.02 (1.01, 1.03) | | | | 0.94 (0.92, 0.96) | | | 0.96 (0.95, 0.98) | | | | 1.05 (1.04, 1.06) | | |  |
|  | High | | 1.25 (1.23, 1.26) | | | | 1.19 (1.17, 1.22) | | | 1.17 (1.15, 1.19) | | | | 1.16 (1.15, 1.17) | | |  |
| Father's education (n=656,321) | |  | |  |  |  | |  |  | |  |  |  | |  |  | |
|  | Basic or less | | (reference) | | | | (reference) | | | (reference) | | | | (reference) | | |  |
|  | Upper secondary | | 1.04 (1.01, 1.06) | | | | 1.05 (1.01, 1.09) | | | 0.99 (0.97, 1.02) | | | | 1.01 (0.99, 1.03) | | |  |
|  | Tertiary or above | | 1.11 (1.08, 1.13) | | | | 1.12 (1.07, 1.16) | | | 1.02 (0.99, 1.05) | | | | 1.10 (1.08, 1.12) | | |  |
| Mother's education (n=663,538) | |  | |  |  |  | |  |  | |  |  |  | |  |  | |
|  | Basic or less | | (reference) | | | | (reference) | | | (reference) | | | | (reference) | | |  |
|  | Upper secondary | | 1.04 (1.02, 1.07) | | | | 1.05 (1.01, 1.10) | | | 1.04 (1.01, 1.07) | | | | 1.03 (1.01, 1.05) | | |  |
|  | Tertiary or above | | 1.16 (1.13, 1.19) | | | | 1.20 (1.15, 1.26) | | | 1.12 (1.08, 1.15) | | | | 1.06 (1.04, 1.08) | | |  |
| Parents' education(n=708,737) | |  | |  |  |  | |  |  | |  |  |  | |  |  | |
|  | Basic or less | | (reference) | | | | (reference) | | | (reference) | | | | (reference) | | |  |
|  | Upper secondary | | 1.06 (1.02, 1.10) | | | | 1.07 (1.00, 1.15) | | | 1.04 (0.99, 1.09) | | | | 1.01 (0.98, 1.04) | | |  |
|  | Tertiary or above | | 1.16 (1.12, 1.20) | | | | 1.19 (1.11, 1.27) | | | 1.09 (1.04, 1.15) | | | | 1.07 (1.04, 1.10) | | |  |
| Urbanization (n=865,614) | |  | |  |  |  | |  |  | |  |  |  | |  |  | |
|  | Rural areas | | (reference) | | | | (reference) | | | (reference) | | | | (reference) | | |  |
|  | Other cities | | 0.93 (0.90, 0.95) | | | | 0.95 (0.91, 0.99) | | | 0.94 (0.91, 0.98) | | | | 0.96 (0.94, 0.98) | | |  |
|  | Metropolitan cities | | 0.94 (0.92, 0.97) | | | | 0.98 (0.94, 1.03) | | | 0.95 (0.92, 0.99) | | | | 0.98 (0.96, 1.00) | | |  |

All statistics were weighted.
PA: physical activity.
