# Supplementary material for: Trends in the effects of socioeconomic position on physical activity levels and sedentary behavior among Korean adolescents
Source: Epidemiol Health. 2023 Sep 8;45:e2023085. doi: 10.4178/epih.e2023085 (PMC10728613; doi:10.4178/epih.e2023085)
Supplement: Supplement Material 3. — Subgroup analysis for sex and school [file epih-45-e2023085-Supplementary-3.docx]

Supplementary Material 3. Subgroup analysis for sex and school

| Variables | | Vigorous PA | | | | Moderate PA | | | | Muscle training | | | | | Sedentary time | | | |
| --- | --- | --- | --- | --- | --- | --- | --- | --- | --- | --- | --- | --- | --- | --- | --- | --- | --- | --- |
|  |  | PR (95% CI) | | | | PR (95% CI) | | | | PR (95% CI) | | | | PR (95% CI) | | | | |
| **Subgroup: Male** | |  |  |  |  |  |  |  |  |  |  |  |  |  | |  |  |  |
| Household income | |  |  |  |  |  |  |  |  |  |  |  |  |  | |  |  |  |
|  | High | 1.21 (1.19, 1.23) | | | | 1.18 (1.14, 1.21) | | | | 1.10 (1.08, 1.12) | | | | 1.15 (1.13, 1.17) | | | | |
|  | Middle | 1.06 (1.04, 1.07) | | | | 0.99 (0.96, 1.01) | | | | 1.00 (0.98, 1.02) | | | | 1.07 (1.06, 1.09) | | | | |
|  | Low | (reference) | | | | (reference) | | | | (reference) | | | | | (reference) | | | |
| Father's education | |  |  |  |  |  |  |  |  |  |  |  |  |  | |  |  |  |
|  | Tertiary or above | 1.15 (1.12, 1.18) | | | | 1.21 (1.15, 1.27) | | | | 1.04 (1.01, 1.08) | | | | 1.14 (1.11, 1.18) | | | | |
|  | Upper secondary | 1.09 (1.06, 1.11) | | | | 1.14 (1.08, 1.20) | | | | 1.04 (1.01, 1.08) | | | | 1.05 (1.02, 1.08) | | | | |
|  | Basic or less | (reference) | | | | (reference) | | | | (reference) | | | | | (reference) | | | |
| Mother's education | |  |  |  |  |  |  |  |  |  |  |  |  |  | |  |  |  |
|  | Tertiary or above | 1.17 (1.14, 1.20) | | | | 1.23 (1.17, 1.30) | | | | 1.11 (1.07, 1.15) | | | | 1.08 (1.05, 1.12) | | | | |
|  | Upper secondary | 1.08 (1.05, 1.11) | | | | 1.11 (1.05, 1.17) | | | | 1.09 (1.05, 1.13) | | | | 1.05 (1.02, 1.08) | | | | |
|  | Basic or less | (reference) | | | | (reference) | | | | (reference) | | | | | (reference) | | | |
| Urbanization | |  |  |  |  |  |  |  |  |  |  |  |  |  | |  |  |  |
|  | Metropolitan cities | 0.96 (0.94, 0.98) | | | | 0.97 (0.93, 1.02) | | | | 0.94 (0.91, 0.96) | | | | 0.98 (0.96, 1.01) | | | | |
|  | Other cities | 0.95 (0.93, 0.98) | | | | 0.95 (0.91, 1.00) | | | | 0.95 (0.92, 0.98) | | | | 0.97 (0.94, 1.00) | | | | |
|  | Rural areas | (reference) | | | | (reference) | | | | (reference) | | | | | (reference) | | | |
| **Subgroup: Female** | |  |  |  |  |  |  |  |  |  |  |  |  |  | |  |  |  |
| Household income | |  |  |  |  |  |  |  |  |  |  |  |  |  | |  |  |  |
|  | High | 1.21 (1.18, 1.24) | | | | 1.07 (1.02, 1.11) | | | | 1.13 (1.09, 1.17) | | | | 1.13 (1.11, 1.15) | | | | |
|  | Middle | 0.99 (0.97, 1.02) | | | | 0.87 (0.84, 0.91) | | | | 0.93 (0.90, 0.97) | | | | 1.04 (1.02, 1.05) | | | | |
|  | Low | (reference) | | | | (reference) | | | | (reference) | | | | | (reference) | | | |
| Father's education | |  |  |  |  |  |  |  |  |  |  |  |  |  | |  |  |  |
|  | Tertiary or above | 1.09 (1.04, 1.14) | | | | 1.03 (0.94, 1.12) | | | | 1.05 (0.98, 1.12) | | | | 1.03 (1.00, 1.06) | | | | |
|  | Upper secondary | 1.04 (0.99, 1.08) | | | | 1.00 (0.92, 1.09) | | | | 1.00 (0.93, 1.07) | | | | 0.96 (0.94, 0.99) | | | | |
|  | Basic or less | (reference) | | | | (reference) | | | | (reference) | | | | | (reference) | | | |
|  |  |  | | | |  | | | |  | | | | | *(Continued)* | | | |

| Variables | | Vigorous PA | | | | Moderate PA | | | | Muscle training | | | | Sedentary time | | | |
| --- | --- | --- | --- | --- | --- | --- | --- | --- | --- | --- | --- | --- | --- | --- | --- | --- | --- |
|  |  | PR (95% CI) | | | | PR (95% CI) | | | | PR (95% CI) | | | | PR (95% CI) | | | |
| Mother's education | |  |  |  |  |  |  |  |  |  |  |  |  |  |  |  |  |
|  | Basic or less | (reference) | | | | (reference) | | | | (reference) | | | | (reference) | | | |
|  | Upper secondary | 1.04 (0.99, 1.09) | | | | 1.03 (0.94, 1.13) | | | | 1.03 (0.96, 1.11) | | | | 1.01 (0.98, 1.04) | | | |
|  | Tertiary or above | 1.14 (1.09, 1.20) | | | | 1.13 (1.04, 1.24) | | | | 1.13 (1.05, 1.21) | | | | 1.02 (0.99, 1.05) | | | |
| Urbanization | |  |  |  |  |  |  |  |  |  |  |  |  |  |  |  |  |
|  | Rural areas | (reference) | | | | (reference) | | | | (reference) | | | | (reference) | | | |
|  | Other cities | 0.91 (0.86, 0.95) | | | | 1.00 (0.92, 1.09) | | | | 0.97 (0.91, 1.03) | | | | 0.95 (0.92, 0.98) | | | |
|  | Metropolitan cities | 0.92 (0.88, 0.97) | | | | 1.05 (0.96, 1.13) | | | | 1.02 (0.95, 1.09) | | | | 0.98 (0.95, 1.01) | | | |
| **Subgroup: High school** | | |  |  |  |  |  |  |  |  |  |  |  |  |  |  |  |
| Household income | |  |  |  |  |  |  |  |  |  |  |  |  |  |  |  |  |
|  | Low | (reference) | | | | (reference) | | | | (reference) | | | | (reference) | | | |
|  | Middle | 0.98 (0.96, 0.99) | | | | 0.89 (0.86, 0.92) | | | | 0.97 (0.95, 1.00) | | | | 1.06 (1.04, 1.07) | | | |
|  | High | 1.15 (1.12, 1.17) | | | | 1.06 (1.02, 1.09) | | | | 1.12 (1.09, 1.15) | | | | 1.12 (1.10, 1.13) | | | |
| Father's education | |  |  |  |  |  |  |  |  |  |  |  |  |  |  |  |  |
|  | Basic or less | (reference) | | | | (reference) | | | | (reference) | | | | (reference) | | | |
|  | Upper secondary | 1.01 (0.98, 1.05) | | | | 1.01 (0.95, 1.07) | | | | 1.00 (0.97, 1.05) | | | | 1.00 (0.98, 1.03) | | | |
|  | Tertiary or above | 1.03 (1.00, 1.07) | | | | 1.00 (0.94, 1.06) | | | | 0.99 (0.95, 1.04) | | | | 1.08 (1.05, 1.10) | | | |
| Mother's education | |  |  |  |  |  |  |  |  |  |  |  |  |  |  |  |  |
|  | Basic or less | (reference) | | | | (reference) | | | | (reference) | | | | (reference) | | | |
|  | Upper secondary | 1.02 (0.99, 1.06) | | | | 1.01 (0.95, 1.07) | | | | 1.05 (1.00, 1.09) | | | | 1.03 (1.01, 1.06) | | | |
|  | Tertiary or above | 1.08 (1.04, 1.12) | | | | 1.04 (0.98, 1.11) | | | | 1.07 (1.02, 1.12) | | | | 1.05 (1.03, 1.08) | | | |
| Urbanization | |  |  |  |  |  |  |  |  |  |  |  |  |  |  |  |  |
|  | Rural areas | (reference) | | | | (reference) | | | | (reference) | | | | (reference) | | | |
|  | Other cities | 0.92 (0.88, 0.96) | | | | 0.93 (0.87, 1.00) | | | | 0.90 (0.85, 0.96) | | | | 0.96 (0.93, 0.99) | | | |
|  | Metropolitan cities | 0.91 (0.87, 0.95) | | | | 0.94 (0.87, 1.01) | | | | 0.88 (0.83, 0.94) | | | | 0.98 (0.95, 1.01) | | | |
| **Subgroup: Middle school** | | |  |  |  |  |  |  |  |  |  |  |  |  |  |  |  |
| Household income | |  |  |  |  |  |  |  |  |  |  |  |  |  |  |  |  |
|  | Low | (reference) | | | | (reference) | | | | (reference) | | | | (reference) | | | |
|  | Middle | 0.98 (0.97, 1.00) | | | | 0.92 (0.89, 0.96) | | | | 0.93 (0.91, 0.96) | | | | 1.10 (1.08, 1.12) | | | |
|  | High | 1.16 (1.14, 1.18) | | | | 1.15 (1.11, 1.19) | | | | 1.15 (1.12, 1.18) | | | | 1.28 (1.25, 1.30) | | | |
|  |  |  | | | |  | | | |  | | | | *(Continued)* | | | |
| Variables | | Vigorous PA | | | | Moderate PA | | | | Muscle training | | | | Sedentary time | | | |
|  |  | PR (95% CI) | | | | PR (95% CI) | | | | PR (95% CI) | | | | PR (95% CI) | | | |
| Father's education | |  |  |  |  |  |  |  |  |  |  |  |  |  |  |  |  |
|  | Basic or less | (reference) | | | | (reference) | | | | (reference) | | | | (reference) | | | |
|  | Upper secondary | 1.01 (0.97, 1.04) | | | | 1.09 (1.01, 1.16) | | | | 0.98 (0.93, 1.03) | | | | 1.05 (1.01, 1.09) | | | |
|  | Tertiary or above | 1.04 (1.01, 1.08) | | | | 1.16 (1.08, 1.24) | | | | 1.02 (0.97, 1.07) | | | | 1.18 (1.13, 1.22) | | | |
| Mother's education | |  |  |  |  |  |  |  |  |  |  |  |  |  |  |  |  |
|  | Basic or less | (reference) | | | | (reference) | | | | (reference) | | | | (reference) | | | |
|  | Upper secondary | 1.00 (0.97, 1.04) | | | | 1.08 (1.01, 1.16) | | | | 1.06 (1.00, 1.12) | | | | 1.05 (1.01, 1.09) | | | |
|  | Tertiary or above | 1.07 (1.03, 1.10) | | | | 1.23 (1.15, 1.32) | | | | 1.15 (1.09, 1.22) | | | | 1.13 (1.09, 1.17) | | | |
| Urbanization | |  |  |  |  |  |  |  |  |  |  |  |  |  |  |  |  |
|  | Rural areas | (reference) | | | | (reference) | | | | (reference) | | | | (reference) | | | |
|  | Other cities | 0.92 (0.88, 0.95) | | | | 0.94 (0.89, 1.00) | | | | 0.96 (0.91, 1.01) | | | | 0.96 (0.93, 0.99) | | | |
|  | Metropolitan cities | 0.95 (0.92, 0.98) | | | | 0.99 (0.94, 1.05) | | | | 1.00 (0.95, 1.05) | | | | 0.98 (0.95, 1.01) | | | |

PA: physical activity; PR: prevalence ratio
All statistics were weighted.
