# Supplementary material for: Trends in the effects of socioeconomic position on physical activity levels and sedentary behavior among Korean adolescents
Source: Epidemiol Health. 2023 Sep 8;45:e2023085. doi: 10.4178/epih.e2023085 (PMC10728613; doi:10.4178/epih.e2023085)
Supplement: Supplement Material 4. — The yearly prevalence ratio between socioeconomical position and physical activity in Korean adolescents [file epih-45-e2023085-Supplementary-4.docx]

Supplementary Material 4. The yearly prevalence ratio between socioeconomical position and physical activity in Korean adolescents

| **Variables** | | **Year** | | | | | | | | | | | | | | | | | | | | | |  |
| --- | --- | --- | --- | --- | --- | --- | --- | --- | --- | --- | --- | --- | --- | --- | --- | --- | --- | --- | --- | --- | --- | --- | --- | --- |
|  |  | **2009** | **2010** | | **2011** | | **2012** | | **2013** | | **2014** | | **2015** | | **2016** | | **2017** | | **2018** | | **2019** | **2020** | **2021** |  |
| **Outcome: Vigorous physical activity** | | | | |  | |  | |  | |  | |  | |  | |  | |  | |  |  |  |  |
| Household income | |  |  | |  | |  | |  | |  | |  | |  | |  | |  | |  |  |  |  |
|  | High | 1.26 | 1.34 | | 1.37 | | 1.28 | | 1.24 | | 1.23 | | 1.17 | | 1.22 | | 1.24 | | 1.20 | | 1.20 | 1.26 | 1.15 |  |
|  |  | (1.20, 1.33) | (1.27, 1.41) | | (1.31, 1.43) | | (1.23, 1.34) | | (1.19, 1.30) | | (1.18, 1.29) | | (1.12, 1.23) | | (1.16, 1.27) | | (1.18, 1.31) | | (1.14, 1.27) | | (1.11, 1.30) | (1.17, 1.36) | (1.07, 1.24) |  |
|  | Middle | 0.98 | 1.04 | | 1.08 | | 1.03 | | 1.02 | | 1.01 | | 0.99 | | 1.03 | | 1.05 | | 1.00 | | 0.95 | 1.01 | 0.95 |  |
|  |  | (0.94, 1.03) | (0.99, 1.09) | | (1.04, 1.12) | | (0.99, 1.07) | | (0.98, 1.06) | | (0.97, 1.06) | | (0.95, 1.03) | | (0.98, 1.07) | | (1.00, 1.10) | | (0.95, 1.05) | | (0.89, 1.03) | (0.93, 1.09) | (0.88, 1.03) |  |
| Father's education | |  |  | |  | |  | |  | |  | |  | |  | |  | |  | |  |  |  |  |
|  | Tertiary or above | 1.13 | 1.10 | | 1.13 | | 1.07 | | 1.11 | | 1.09 | | 1.18 | | 1.12 | | 1.17 | | 1.06 | | 1.05 | 1.16 | 0.93 |  |
|  |  | (1.06, 1.21) | (1.02, 1.18) | | (1.06, 1.21) | | (1.00, 1.16) | | (1.03, 1.19) | | (1.01, 1.18) | | (1.08, 1.29) | | (1.02, 1.24) | | (1.06, 1.30) | | (0.95, 1.19) | | (0.89, 1.24) | (0.97, 1.39) | (0.80, 1.08) |  |
|  | Upper secondary | 1.06 | 1.01 | | 1.03 | | 0.98 | | 1.04 | | 1.01 | | 1.10 | | 1.07 | | 1.08 | | 1.00 | | 1.02 | 1.18 | 0.93 |  |
|  |  | (0.99, 1.13) | (0.94, 1.08) | | (0.97, 1.10) | | (0.91, 1.06) | | (0.97, 1.12) | | (0.94, 1.08) | | (1.01, 1.20) | | (0.97, 1.18) | | (0.98, 1.19) | | (0.89, 1.12) | | (0.86, 1.20) | (0.99, 1.41) | (0.80, 1.09) |  |
| Mother's education | |  |  | |  | |  | |  | |  | |  | |  | |  | |  | |  |  |  |  |
|  | Tertiary or above | 1.17 | 1.14 | | 1.23 | | 1.17 | | 1.15 | | 1.17 | | 1.19 | | 1.15 | | 1.13 | | 1.18 | | 1.18 | 1.24 | 0.99 |  |
|  |  | (1.09, 1.26) | (1.05, 1.23) | | (1.15, 1.32) | | (1.09, 1.26) | | (1.07, 1.24) | | (1.06, 1.28) | | (1.07, 1.31) | | (1.04, 1.27) | | (1.01, 1.27) | | (1.03, 1.35) | | (0.97, 1.43) | (1.00, 1.52) | (0.82, 1.19) |  |
|  | Upper secondary | 1.02 | 0.99 | | 1.06 | | 1.04 | | 1.04 | | 1.08 | | 1.08 | | 1.03 | | 1.02 | | 1.08 | | 1.11 | 1.20 | 0.94 |  |
|  |  | (0.96, 1.10) | (0.92, 1.06) | | (0.99, 1.13) | | (0.97, 1.12) | | (0.97, 1.11) | | (0.99, 1.18) | | (0.98, 1.19) | | (0.94, 1.14) | | (0.91, 1.14) | | (0.94, 1.24) | | (0.91, 1.34) | (0.98, 1.48) | (0.78, 1.14) |  |
| Urbanicity | |  |  | |  | |  | |  | |  | |  | |  | |  | |  | |  |  |  |  |
|  | Metropolitan cities | 0.95 | 0.94 | | 1.01 | | 0.96 | | 0.95 | | 0.92 | | 0.92 | | 0.89 | | 0.99 | | 0.92 | | 0.97 | 0.89 | 0.87 |  |
|  |  | (0.88, 1.03) | (0.86, 1.02) | | (0.93, 1.10) | | (0.87, 1.05) | | (0.86, 1.04) | | (0.83, 1.02) | | (0.82, 1.03) | | (0.78, 1.01) | | (0.87, 1.13) | | (0.82, 1.04) | | (0.83, 1.14) | (0.78, 1.01) | (0.77, 0.97) |  |
|  | Other cities | 0.92 | 0.91 | | 0.99 | | 0.94 | | 0.92 | | 0.91 | | 0.90 | | 0.90 | | 0.98 | | 0.92 | | 0.94 | 0.88 | 0.89 |  |
|  |  | (0.85, 1.01) | (0.83, 1.00) | | (0.91, 1.09) | | (0.85, 1.03) | | (0.84, 1.01) | | (0.82, 1.00) | | (0.80, 1.01) | | (0.79, 1.01) | | (0.86, 1.11) | | (0.82, 1.03) | | (0.80, 1.10) | (0.78, 1.00) | (0.79, 1.00) |  |
|  |  |  |  | |  | |  | |  | |  | |  | |  | |  | |  | |  | *(Continued)* | |  |
| **Variables** | | **Year** | | | | | | | | | | | | | | | | | | | | | |  |
|  |  | **2009** | | **2010** | | **2011** | | **2012** | | **2013** | | **2014** | | **2015** | | **2016** | | **2017** | | **2018** | **2019** | **2020** | **2021** |  |
| **Outcome: Moderate physical activity** | | | | | |  | |  | |  | |  | |  | |  | |  | |  |  |  |  |  |
| Household income | |  | |  | |  | |  | |  | |  | |  | |  | |  | |  |  |  |  |  |
|  | High | 1.22 | | 1.12 | | 1.24 | | 1.24 | | 1.19 | | 1.16 | | 1.15 | | 1.13 | | 1.03 | | 1.08 | 1.15 | 1.19 | 1.03 |  |
|  |  | (1.12, 1.32) | | (1.02, 1.23) | | (1.15, 1.34) | | (1.16, 1.34) | | (1.10, 1.29) | | (1.08, 1.25) | | (1.07, 1.25) | | (1.03, 1.23) | | (0.95, 1.13) | | (0.99, 1.18) | (1.02, 1.29) | (1.07, 1.33) | (0.93, 1.15) |  |
|  | Middle | 0.85 | | 0.83 | | 0.91 | | 0.98 | | 0.92 | | 0.95 | | 0.96 | | 0.95 | | 0.91 | | 0.87 | 0.92 | 0.96 | 0.83 |  |
|  |  | (0.79, 0.92) | | (0.76, 0.91) | | (0.84, 0.98) | | (0.91, 1.06) | | (0.85, 0.99) | | (0.89, 1.02) | | (0.89, 1.03) | | (0.87, 1.03) | | (0.83, 0.99) | | (0.80, 0.95) | (0.82, 1.04) | (0.86, 1.08) | (0.75, 0.93) |  |
| Father's education | |  | |  | |  | |  | |  | |  | |  | |  | |  | |  |  |  |  |  |
|  | Tertiary or above | 1.11 | | 1.03 | | 1.13 | | 1.08 | | 1.22 | | 1.00 | | 1.06 | | 1.00 | | 1.14 | | 1.07 | 1.11 | 1.10 | 0.90 |  |
|  |  | (0.99, 1.25) | | (0.89, 1.19) | | (0.99, 1.28) | | (0.95, 1.22) | | (1.05, 1.42) | | (0.88, 1.15) | | (0.91, 1.24) | | (0.84, 1.19) | | (0.94, 1.37) | | (0.86, 1.32) | (0.85, 1.43) | (0.85, 1.42) | (0.69, 1.18) |  |
|  | Upper secondary | 1.00 | | 0.95 | | 1.02 | | 1.01 | | 1.13 | | 0.96 | | 1.04 | | 0.98 | | 1.15 | | 1.10 | 1.16 | 1.12 | 0.96 |  |
|  |  | (0.89, 1.13) | | (0.82, 1.10) | | (0.90, 1.16) | | (0.88, 1.15) | | (0.97, 1.32) | | (0.84, 1.10) | | (0.89, 1.21) | | (0.82, 1.16) | | (0.94, 1.39) | | (0.88, 1.37) | (0.89, 1.50) | (0.86, 1.45) | (0.73, 1.27) |  |
| Mother's education | |  | |  | |  | |  | |  | |  | |  | |  | |  | |  |  |  |  |  |
|  | Tertiary or above | 1.21 | | 1.11 | | 1.19 | | 1.19 | | 1.14 | | 1.11 | | 1.23 | | 1.03 | | 1.13 | | 1.05 | 1.15 | 1.02 | 0.89 |  |
|  |  | (1.07, 1.38) | | (0.97, 1.28) | | (1.04, 1.37) | | (1.05, 1.36) | | (0.99, 1.32) | | (0.96, 1.29) | | (1.03, 1.46) | | (0.86, 1.25) | | (0.91, 1.39) | | (0.81, 1.34) | (0.82, 1.60) | (0.75, 1.38) | (0.67, 1.18) |  |
|  | Upper secondary | 0.97 | | 0.95 | | 1.01 | | 1.06 | | 1.01 | | 1.04 | | 1.15 | | 0.96 | | 1.09 | | 1.01 | 1.14 | 1.01 | 0.94 |  |
|  |  | (0.86, 1.10) | | (0.84, 1.09) | | (0.89, 1.16) | | (0.94, 1.20) | | (0.88, 1.17) | | (0.90, 1.20) | | (0.96, 1.37) | | (0.79, 1.16) | | (0.89, 1.35) | | (0.78, 1.30) | (0.82, 1.59) | (0.75, 1.37) | (0.70, 1.25) |  |
| Urbanicity | |  | |  | |  | |  | |  | |  | |  | |  | |  | |  |  |  |  |  |
|  | Metropolitan cities | 1.14 | | 0.93 | | 1.06 | | 1.01 | | 1.02 | | 0.96 | | 0.88 | | 0.86 | | 1.04 | | 0.92 | 0.98 | 1.03 | 0.88 |  |
|  |  | (0.99, 1.31) | | (0.82, 1.07) | | (0.94, 1.19) | | (0.89, 1.14) | | (0.89, 1.17) | | (0.85, 1.10) | | (0.72, 1.09) | | (0.66, 1.11) | | (0.84, 1.30) | | (0.78, 1.10) | (0.79, 1.21) | (0.86, 1.22) | (0.77, 1.01) |  |
|  | Other cities | 1.06 | | 0.89 | | 1.02 | | 0.98 | | 0.96 | | 0.92 | | 0.85 | | 0.86 | | 1.02 | | 0.90 | 0.93 | 1.01 | 0.85 |  |
|  |  | (0.91, 1.23) | | (0.77, 1.03) | | (0.91, 1.15) | | (0.86, 1.11) | | (0.84, 1.10) | | (0.81, 1.05) | | (0.69, 1.04) | | (0.66, 1.11) | | (0.82, 1.27) | | (0.76, 1.07) | (0.75, 1.15) | (0.85, 1.20) | (0.75, 0.98) |  |
|  |  |  | |  | |  | |  | |  | |  | |  | |  | |  | |  |  | *(Continued)* | |  |

| **Variables** | | **Year** | | | | | | | | | | | | |
| --- | --- | --- | --- | --- | --- | --- | --- | --- | --- | --- | --- | --- | --- | --- |
|  |  | **2009** | **2010** | **2011** | **2012** | **2013** | **2014** | **2015** | **2016** | **2017** | **2018** | **2019** | **2020** | **2021** |
| **Outcome: Muscle training** | | |  |  |  |  |  |  |  |  |  |  |  |  |
| Household income | |  |  |  |  |  |  |  |  |  |  |  |  |  |
|  | High | 1.20 | 1.22 | 1.30 | 1.14 | 1.16 | 1.09 | 1.00 | 1.07 | 1.10 | 1.13 | 1.07 | 1.20 | 1.03 |
|  |  | (1.13, 1.28) | (1.15, 1.30) | (1.23, 1.38) | (1.08, 1.21) | (1.09, 1.22) | (1.02, 1.16) | (0.94, 1.07) | (1.00, 1.15) | (1.03, 1.18) | (1.06, 1.22) | (0.97, 1.17) | (1.11, 1.31) | (0.95, 1.12) |
|  | Middle | 0.98 | 0.97 | 1.03 | 0.98 | 0.95 | 0.95 | 0.87 | 0.91 | 0.94 | 0.96 | 0.89 | 1.00 | 0.85 |
|  |  | (0.93, 1.04) | (0.92, 1.02) | (0.98, 1.08) | (0.93, 1.03) | (0.90, 1.00) | (0.90, 1.01) | (0.82, 0.93) | (0.86, 0.97) | (0.88, 1.00) | (0.90, 1.03) | (0.82, 0.98) | (0.93, 1.09) | (0.78, 0.93) |
| Father's education | |  |  |  |  |  |  |  |  |  |  |  |  |  |
|  | Tertiary or above | 1.09 | 1.01 | 1.03 | 1.01 | 1.11 | 1.01 | 1.02 | 0.85 | 0.96 | 0.90 | 0.77 | 1.03 | 0.79 |
|  |  | (1.00, 1.19) | (0.93, 1.10) | (0.94, 1.13) | (0.92, 1.11) | (1.00, 1.24) | (0.91, 1.11) | (0.90, 1.16) | (0.76, 0.97) | (0.84, 1.10) | (0.76, 1.07) | (0.63, 0.93) | (0.85, 1.24) | (0.66, 0.94) |
|  | Upper secondary | 1.03 | 0.96 | 0.96 | 0.98 | 1.09 | 0.99 | 1.03 | 0.86 | 0.98 | 0.93 | 0.82 | 1.07 | 0.84 |
|  |  | (0.95, 1.12) | (0.88, 1.04) | (0.87, 1.05) | (0.90, 1.08) | (0.98, 1.21) | (0.90, 1.09) | (0.91, 1.16) | (0.76, 0.97) | (0.85, 1.12) | (0.79, 1.11) | (0.68, 0.99) | (0.89, 1.29) | (0.70, 1.00) |
| Mother's education | |  |  |  |  |  |  |  |  |  |  |  |  |  |
|  | Tertiary or above | 1.17 | 1.16 | 1.11 | 1.14 | 1.11 | 1.07 | 1.20 | 0.91 | 1.04 | 1.08 | 0.98 | 0.92 | 0.96 |
|  |  | (1.06, 1.29) | (1.05, 1.27) | (1.01, 1.21) | (1.03, 1.26) | (0.99, 1.24) | (0.95, 1.20) | (1.04, 1.39) | (0.80, 1.04) | (0.89, 1.22) | (0.89, 1.30) | (0.78, 1.23) | (0.76, 1.11) | (0.76, 1.21) |
|  | Upper secondary | 1.05 | 1.05 | 0.98 | 1.06 | 1.03 | 1.04 | 1.17 | 0.89 | 1.05 | 1.07 | 1.03 | 0.88 | 0.98 |
|  |  | (0.97, 1.15) | (0.96, 1.15) | (0.90, 1.07) | (0.96, 1.17) | (0.92, 1.15) | (0.93, 1.17) | (1.02, 1.35) | (0.78, 1.02) | (0.90, 1.23) | (0.88, 1.30) | (0.83, 1.28) | (0.73, 1.07) | (0.77, 1.24) |
| Urbanicity | |  |  |  |  |  |  |  |  |  |  |  |  |  |
|  | Metropolitan cities | 1.09 | 0.92 | 0.94 | 0.99 | 0.96 | 0.94 | 0.93 | 0.83 | 0.97 | 0.85 | 0.97 | 0.90 | 0.87 |
|  |  | (0.97, 1.21) | (0.83, 1.02) | (0.85, 1.04) | (0.88, 1.11) | (0.84, 1.09) | (0.82, 1.07) | (0.79, 1.10) | (0.68, 1.02) | (0.82, 1.15) | (0.73, 1.00) | (0.82, 1.15) | (0.77, 1.06) | (0.74, 1.02) |
|  | Other cities | 1.02 | 0.92 | 0.90 | 0.98 | 0.93 | 0.95 | 0.90 | 0.84 | 0.97 | 0.89 | 0.94 | 0.93 | 0.92 |
|  |  | (0.91, 1.16) | (0.82, 1.03) | (0.80, 1.00) | (0.87, 1.10) | (0.82, 1.06) | (0.84, 1.09) | (0.76, 1.06) | (0.68, 1.03) | (0.82, 1.15) | (0.76, 1.04) | (0.79, 1.11) | (0.79, 1.08) | (0.78, 1.07) |
|  |  |  |  |  |  |  |  |  |  |  |  |  | *(Continued)* | |

| **Variables** | | **Year** | | | | | | | | | | | | |
| --- | --- | --- | --- | --- | --- | --- | --- | --- | --- | --- | --- | --- | --- | --- |
|  |  | **2009** | **2010** | **2011** | **2012** | **2013** | **2014** | **2015** | **2016** | **2017** | **2018** | **2019** | **2020** | **2021** |
| **Outcome: Sedentary time** | | |  |  |  |  |  |  |  |  |  |  |  |  |
| Household income | |  |  |  |  |  |  |  |  |  |  |  |  |  |
|  | High | 1.31 | 1.31 | 1.37 | 1.27 | 1.25 | 1.20 | 1.16 | 1.15 | 1.14 | 1.28 | 1.20 | 1.16 | 1.25 |
|  |  | (1.28, 1.35) | (1.27, 1.34) | (1.33, 1.42) | (1.24, 1.31) | (1.21, 1.30) | (1.16, 1.25) | (1.11, 1.21) | (1.10, 1.20) | (1.09, 1.20) | (1.20, 1.36) | (1.12, 1.30) | (1.06, 1.27) | (1.14, 1.37) |
|  | Middle | 1.16 | 1.17 | 1.19 | 1.12 | 1.11 | 1.06 | 1.07 | 1.04 | 1.04 | 1.10 | 1.06 | 0.97 | 1.06 |
|  |  | (1.13, 1.19) | (1.13, 1.20) | (1.15, 1.22) | (1.09, 1.15) | (1.07, 1.15) | (1.02, 1.10) | (1.03, 1.12) | (1.00, 1.08) | (0.99, 1.09) | (1.03, 1.17) | (0.98, 1.14) | (0.88, 1.06) | (0.97, 1.16) |
| Father's education | |  |  |  |  |  |  |  |  |  |  |  |  |  |
|  | Tertiary or above | 1.36 | 1.30 | 1.28 | 1.26 | 1.20 | 1.18 | 1.11 | 1.00 | 1.17 | 1.33 | 1.37 | 1.05 | 1.11 |
|  |  | (1.30, 1.42) | (1.24, 1.35) | (1.22, 1.35) | (1.20, 1.32) | (1.12, 1.28) | (1.09, 1.27) | (1.03, 1.20) | (0.93, 1.08) | (1.05, 1.29) | (1.14, 1.54) | (1.15, 1.65) | (0.84, 1.30) | (0.90, 1.37) |
|  | Upper secondary | 1.12 | 1.09 | 1.06 | 1.08 | 1.07 | 1.06 | 1.02 | 0.93 | 1.03 | 1.13 | 1.22 | 0.91 | 0.95 |
|  |  | (1.08, 1.18) | (1.04, 1.14) | (1.01, 1.11) | (1.03, 1.14) | (1.00, 1.14) | (0.98, 1.14) | (0.94, 1.11) | (0.86, 1.00) | (0.93, 1.14) | (0.97, 1.31) | (1.01, 1.47) | (0.73, 1.14) | (0.77, 1.17) |
| Mother's education | |  |  |  |  |  |  |  |  |  |  |  |  |  |
|  | Tertiary or above | 1.37 | 1.34 | 1.30 | 1.25 | 1.22 | 1.21 | 1.11 | 1.10 | 1.12 | 1.20 | 1.14 | 1.20 | 1.13 |
|  |  | (1.31, 1.44) | (1.27, 1.40) | (1.23, 1.38) | (1.19, 1.31) | (1.14, 1.31) | (1.11, 1.32) | (1.02, 1.21) | (1.00, 1.21) | (1.01, 1.23) | (1.02, 1.41) | (0.96, 1.35) | (0.91, 1.58) | (0.89, 1.42) |
|  | Upper secondary | 1.16 | 1.15 | 1.09 | 1.10 | 1.09 | 1.10 | 1.01 | 1.02 | 1.00 | 1.01 | 1.02 | 1.06 | 0.98 |
|  |  | (1.11, 1.21) | (1.10, 1.20) | (1.03, 1.15) | (1.06, 1.15) | (1.02, 1.17) | (1.01, 1.19) | (0.93, 1.10) | (0.93, 1.13) | (0.90, 1.10) | (0.86, 1.19) | (0.86, 1.22) | (0.81, 1.39) | (0.78, 1.24) |
| Urbanicity | |  |  |  |  |  |  |  |  |  |  |  |  |  |
|  | Metropolitan cities | 1.00 | 0.99 | 1.03 | 0.98 | 0.96 | 0.96 | 0.90 | 0.95 | 0.95 | 0.98 | 1.00 | 0.82 | 1.02 |
|  |  | (0.95, 1.06) | (0.94, 1.04) | (0.97, 1.08) | (0.93, 1.04) | (0.89, 1.03) | (0.90, 1.03) | (0.83, 0.97) | (0.88, 1.03) | (0.87, 1.04) | (0.90, 1.08) | (0.92, 1.09) | (0.73, 0.93) | (0.92, 1.13) |
|  | Other cities | 1.07 | 1.02 | 1.00 | 0.96 | 0.97 | 0.93 | 0.87 | 0.96 | 0.91 | 0.94 | 1.03 | 0.84 | 0.98 |
|  |  | (1.01, 1.14) | (0.97, 1.08) | (0.95, 1.06) | (0.91, 1.02) | (0.90, 1.04) | (0.87, 1.00) | (0.81, 0.94) | (0.89, 1.04) | (0.83, 0.99) | (0.86, 1.04) | (0.94, 1.12) | (0.74, 0.95) | (0.89, 1.09) |
|  |  |  |  |  |  |  |  |  |  |  |  |  |  | |
